# ChatGPT’s Accuracy on Magnetic Resonance Imaging Basics: Characteristics and Limitations Depending on the Question Type

**DOI:** 10.3390/diagnostics14020171

**Published:** 2024-01-12

**Authors:** Kyu-Hong Lee, Ro-Woon Lee

**Affiliations:** Department of Radiology, Inha University College of Medicine, Incheon 22212, Republic of Korea; mdcappuccino@daum.net

**Keywords:** ChatGPT, MCQ, MRI

## Abstract

Our study aimed to assess the accuracy and limitations of ChatGPT in the domain of MRI, focused on evaluating ChatGPT’s performance in answering simple knowledge questions and specialized multiple-choice questions related to MRI. A two-step approach was used to evaluate ChatGPT. In the first step, 50 simple MRI-related questions were asked, and ChatGPT’s answers were categorized as correct, partially correct, or incorrect by independent researchers. In the second step, 75 multiple-choice questions covering various MRI topics were posed, and the answers were similarly categorized. The study utilized Cohen’s kappa coefficient for assessing interobserver agreement. ChatGPT demonstrated high accuracy in answering straightforward MRI questions, with over 85% classified as correct. However, its performance varied significantly across multiple-choice questions, with accuracy rates ranging from 40% to 66.7%, depending on the topic. This indicated a notable gap in its ability to handle more complex, specialized questions requiring deeper understanding and context. In conclusion, this study critically evaluates the accuracy of ChatGPT in addressing questions related to Magnetic Resonance Imaging (MRI), highlighting its potential and limitations in the healthcare sector, particularly in radiology. Our findings demonstrate that ChatGPT, while proficient in responding to straightforward MRI-related questions, exhibits variability in its ability to accurately answer complex multiple-choice questions that require more profound, specialized knowledge of MRI. This discrepancy underscores the nuanced role AI can play in medical education and healthcare decision-making, necessitating a balanced approach to its application.

## 1. Introduction

Artificial intelligence (AI) has made significant advancements in various fields, including healthcare, finance, and transportation. In healthcare, AI has particularly shown promise in the field of medical imaging. AI-powered algorithms can assist radiologists in detecting abnormalities, segmenting structures, and classifying images with high accuracy [1]. For example, AI-based systems have been developed for automated diagnosis of conditions such as breast cancer, lung nodules, and retinal diseases [2]. AI can also aid in personalized treatment planning and monitoring disease progression, leading to improved healthcare outcomes and reduced costs [3].

In recent years, there has been significant development in medical AI models that assist healthcare professionals in diagnosis, treatment planning, and disease monitoring. These models utilize machine learning algorithms to analyze large volumes of medical data and generate predictions based on identified patterns and trends. Convolutional neural networks (CNNs) have proven effective in detecting abnormalities in medical images such as X-rays, CT scans, and MRIs [4]. Other models, like recurrent neural networks (RNNs) and support vector machines (SVMs), have also been utilized in medical AI applications [2]. These AI models have the potential to enhance diagnostic accuracy, improve treatment planning, and enable real-time monitoring of disease progression, ultimately leading to better patient outcomes [5].

However, there are limitations to label-based learning, which is the most commonly used approach in medical AI. Label-based learning involves training the model using labeled data, where each data point is assigned a specific diagnosis or classification. This approach can be limited by the availability and quality of labeled data, as well as the potential for bias in the labeling process. As a result, there is a need for more advanced AI models that can learn from unlabeled data and improve their predictions over time.

ChatGPT, developed by OpenAI (San Francisco, CA, USA), is a significant language generation model that has shown remarkable abilities in natural language processing, knowledge retrieval, and text generation. It has the potential to be a valuable tool in healthcare, assisting healthcare professionals in answering clinical questions, providing patient education, and facilitating communication between healthcare providers and patients. The model’s language learning module (LLM) allows it to continually improve its performance and accuracy by learning from new data and adapting to new contexts. While there are challenges and limitations in using ChatGPT and other language generation models in healthcare, their potential benefits are significant and warrant further exploration [6].

ChatGPT has shown promise in improving the accuracy and efficiency of radiological diagnoses, reducing interpretation variability and errors, and improving workflow efficiency [7]. However, its performance is contingent on the type of question posed, excelling in tasks requiring lower order thinking and clinical management but struggling with more complex tasks such as calculations, classifications, and the application of advanced concepts [8]. The model also performed better on questions related to lower-order thinking and clinical management compared to higher-order thinking questions involving description of imaging findings, calculation and classification, and application of concepts [9]. Additionally, the model’s performance on physics-related questions was poorer, which is concerning given the importance of radiological physics in the field [9]. Especially in the clinical use of MRI, an understanding of MRI physics can influence the choice of protocols and improve image quality. Understanding and training in this area are essential for radiologists and clinicians, so analyzing the potential use of ChatGPT in this area has implications for the clinical use of MRI.

In this study, we aimed to assess the accuracy and limitations of ChatGPT, a large language generation model, in the domain of MRI physics, the fine art of radiology. Specifically, to evaluate its performance in the field of MRI, we designed a two-step approach where we asked ChatGPT questions and analyzed the answers for accuracy and consistency.

## 2. Materials and Method

### 2.1. Artificial Intelligence

We used the GPT-4 version (paid plan) of ChatGPT (OpenAI, San Francisco, CA, USA) for this study; we did not use any additional plugins to evaluate the problem-solving performance of this large language model on its own. We further protected the information during the question input process by turning off the “Chat History and Training” option in ChatGPT. This setting ensures that conversations and images shared during the session are not used for further training of the AI model or accessed in future sessions. This process is a precautionary measure to ensure that no residual or indirect information is used in a way that could infringe on the rights of the question copyright holder.

### 2.2. Input Source

We envisioned this study as a two-stage questioning process. The initial stage involved posing simple, knowledge-based questions related to MRI. Two experienced researchers (K.H.L and R.W.L), each with 10 years of expertise in MRI physics and MR image interpretation, were engaged to formulate these questions. The researchers selected 50 MRI-related questions to be posed to ChatGPT (Text S1). These questions were meticulously chosen based on several criteria to ensure their relevance, clarity, and objectivity. The criteria included: 1. Relevance to MRI Physics and Clinical Application- Questions were designed to cover essential aspects of MRI, from technical specifications to regulatory standards and historical milestones in MRI development. 2. Unambiguity- Each question was framed to be straightforward and clear, minimizing the potential for multiple interpretations. 3. Non-controversial Nature- The questions were intentionally non-controversial to avoid subjective or debate-prone topics, focusing instead on factual, established knowledge in the field of MRI.

Such questions included “What is the highest magnetic field strength that the U.S. Food and Drug Administration (FDA) allows adults in routine clinical practice?” and “What is the first company to produce a clinical whole-body MRI scanner for commercial use?”.

The initial set of questions was discussed and reviewed by both researchers, and 50 short-answer questions that met the criteria were finally selected. This question selection process aimed to create a standardized set of questions that would objectively assess ChatGPT’s accuracy and reliability in providing information on MRI-related topics.

**Figure 1:**
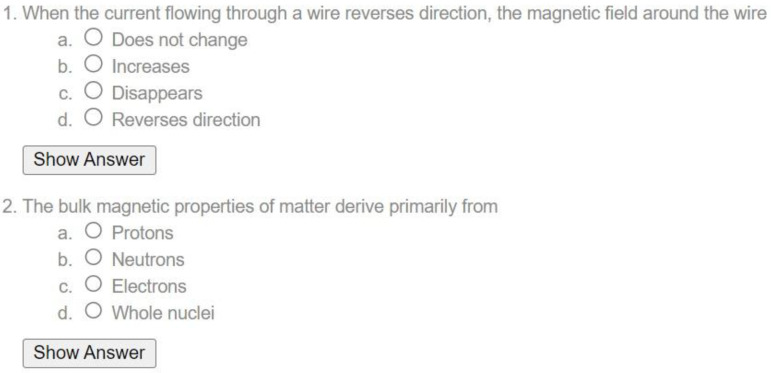
Example of multichoice questions (MCQs).

In the second step, the multiple-choice questions were based on knowledge related to the basics and physics of MRI. In addition to ensuring the professionalism of the questions in ChatGPT, we chose questions from educational sites that have released questions to the public to avoid copyright violations (Text S1). Based on these considerations, the questions were extracted from the public MR study website (Courtesy of Allen D. Elster, MRIquestions.com (accessed on 29 October 2023)). Note that the creator of this site gives permission to use the questions posted on the site for academic purposes. Each question is a multiple-choice question with a single exact answer (Figure 1). The extracted questions were categorized into Basic Electromagnetism, MR Magnets, Gradients, RF and Coils, and Site Planning, and only 15 questions were selected and assigned to each category. The selection of these questions was performed by consensus among the researchers.

### 2.3. Questioning Process for Study

1. Simple questions about magnetic resonance imaging

**Figure 2:**
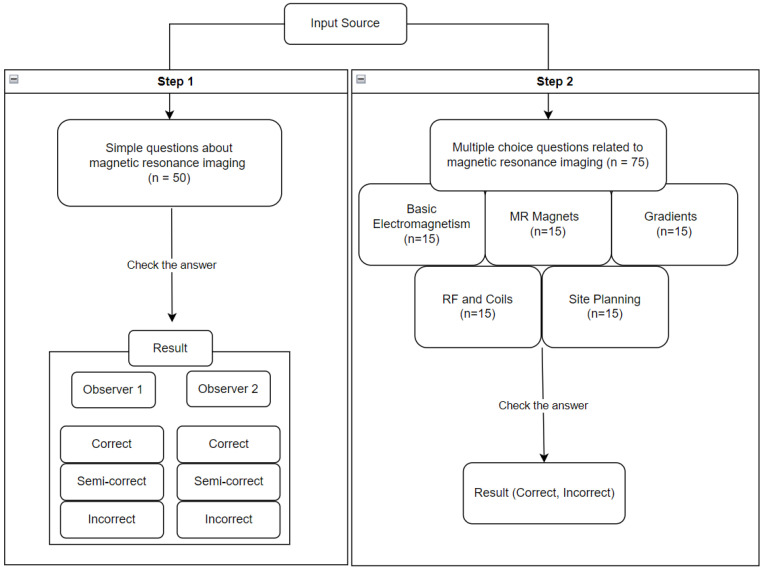
Processes of data input and analysis.

The 50 questions set by the researchers were entered into ChatGPT, and the answers were checked. Two researchers (K.H.L and R.W.L) categorized the answers as “correct”, “partially correct”, and “incorrect”. The classification was performed in an independent session without interference from each other (Figure 2). A correct answer was defined as an answer generated by ChatGPT that accurately described the expert knowledge without apparent errors. This correct answer was agreed upon by both researchers, and the preliminary question selection process ensured that the questions were clear and uncontroversial. A partially correct answer was defined as an answer that was largely accurate but contained at least one error. An incorrect answer was defined as an answer that needed to contain accurate information.

2. Multiple choice questions related to musculoskeletal imaging

A total of 75 questions were entered into ChatGPT, one by one, for each of the three pre-extracted MR domains (Basic Electromagnetism, MR Magnets, Gradients, RF and Coils, and Site Planning). The questions were entered in random order, regardless of discipline. The resulting answers were checked and categorized into correct and incorrect answers (Figure 2). The correct answers to the multiple-choice questions followed the answers on the website (Courtesy of Allen D. Elster, MRIquestions.com (accessed on 29 October 2023)) that provided the questions, and both researchers verified these answers through textbook and web searches to ensure that they were correct. Incorrect answers were defined as answers with a limited interpretation or answers that did not indicate a clear, correct answer.

### 2.4. Statistics

In step 1, we categorized the correct, partially correct, and incorrect answers to the questions by an observer and checked the interobserver agreement through Cohen’s kappa coefficient (Poor = Less than 0.2; Fair = 0.2 to 0.4; Moderate = 0.4 to 0.6; Substantial = 0.6 to 0.8; Near Perfect = 0.8 to 0.99; and Perfect = 1). In step 2, we checked the correct answers generated for each question, categorized them into accurate and incorrect answers, and calculated the correctness rate by dividing the correct answers for each item by the total number of questions. This study utilized the latest version of Python for data analysis (version 3.12.0).

## 3. Result and Discussion

### 3.1. Simple Questions about MRI

In the study, out of 50 questions, Observer 1 found 43 (86%) correct, 5 (10%) semi-correct, and 2 (4%) incorrect, whereas Observer 2 found 44 (88%) correct, 5 (10%) semi-correct, and 1 (2%) incorrect. The interobserver agreement was high, with a Cohen’s kappa coefficient of 0.87, indicating near-perfect agreement (Table 1).

An in-depth analysis of the incorrect and semi-correct answers revealed that ChatGPT tended to struggle with questions requiring highly specific physics knowledge or recent updates in MRI protocols. However, it excelled in questions based on fundamental MRI principles and historical data.

### 3.2. Multiple Choice Questions Related to MRI

We also assessed ChatGPT’s performance on multiple-choice questions related to musculoskeletal imaging across five categories: Basic Electromagnetism, MR Magnets, Gradients, RF and Coils, and Site Planning. The model’s correct answer rates varied across these categories, with 10/15 in Basic Electromagnetism, 8/15 in MR Magnets, 7/15 in Gradients, 9/15 in RF and Coils, and 6/15 in Site Planning.

Further analysis of the incorrect responses in the multiple-choice section indicated that ChatGPT’s performance was inconsistent in areas requiring up-to-date clinical knowledge or intricate details about advanced MRI technology. Notably, it demonstrated a stronger grasp in foundational topics such as Basic Electromagnetism but was less accurate in more complex categories like Gradients and RF and Coils.

The overall accuracy rates in these categories were 66.7%, 53.3%, 46.7%, 40%, and 60%, respectively (Table 2).

**Figure 3:**
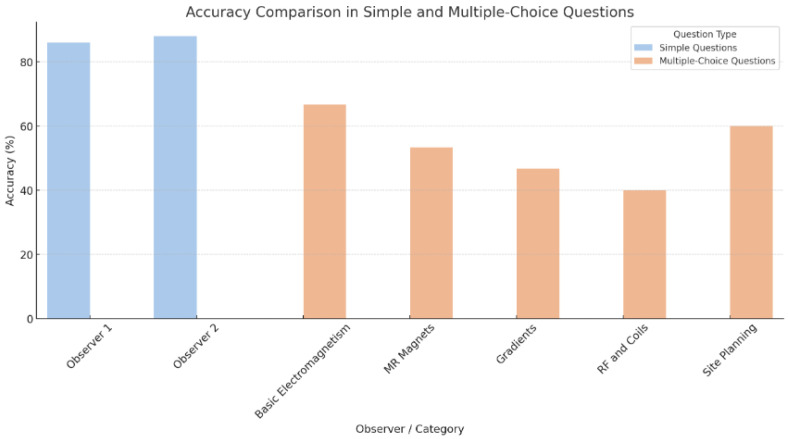
Accuracy Comparison in Simple and Multiple-Choice Questions.

The analysis results demonstrate that while ChatGPT shows high accuracy in answering straightforward MRI questions, its performance on specialized (Figure 3) and multiple-choice questions is more variable, highlighting potential limitations in its application to clinical and educational settings in radiology.

### 3.3. Discussion

In this study, we analyzed the accuracy of ChatGPT in the MRI domain. We found that ChatGPT was generally accurate in answering simple, well-defined questions but needed to be more able to solve off-the-beaten-path multiple-choice questions that required specialized MRI knowledge and judgment.

ChatGPT, developed by OpenAI, is a large-scale language generation model that has been trained on a large corpus of text data. It uses a deep neural network with self-attention mechanisms and a transformer-based architecture to understand natural language and generate contextually appropriate and linguistically coherent responses. The success of ChatGPT can be attributed to its large number of parameters and its ability to learn from vast amounts of data. It is well-suited for complex language tasks due to its ability to handle long-range dependencies [10].

ChatGPT has shown significant potential in the healthcare domain as a tool for generating natural language responses to medical questions and assisting healthcare professionals in decision-making. It has been pre-trained on a large corpus of text data, allowing it to learn a wide range of linguistic patterns and structures. Additionally, the model has been fine-tuned on specialized medical text data, improving its accuracy in answering medical questions. Its ability to generate accurate and contextually appropriate responses has the potential to improve patient outcomes and increase efficiency in healthcare settings [11].

The integration of Large Language Models (LLMs) like ChatGPT into clinical practice, particularly in radiology, brings both advantages and challenges. On the positive side, LLMs offer advanced natural language processing capabilities, enabling tasks such as text summarization, translation, and question-answering, which can be particularly useful in interpreting and communicating complex radiological reports in a patient-friendly language. Additionally, LLMs can be instrumental in generating code for medical imaging research, and, when combined with convolutional neural networks (CNNs), they can assist in image recognition and relevant text generation, enhancing research in medical image analysis.

However, several risks and difficulties are inherent in the use of LLMs in clinical settings. One major concern is privacy, as sensitive patient information could be compromised when uploaded to LLMs, raising serious ethical concerns [12]. Moreover, LLMs may generate artificial or potentially harmful information, such as incorrect translations or diagnostic conclusions, which necessitates thorough validation of LLM-generated content, particularly in patient care.

Another challenge is the interpretability and transparency of LLMs [12]. It is crucial for medical professionals to understand why a model produces a certain output, especially when these outputs have a direct impact on patient care. The accuracy of LLM outputs is heavily dependent on the quality and diversity of the training data. Generic models not specifically trained on medical data might provide inaccurate responses to medical tasks [13]. Even medically oriented LLMs could have limitations in their representation of certain information [13]. Additionally, the rapid evolution of medical knowledge poses a challenge, as LLMs might not have access to the latest data and guidelines.

**Figure 4:**
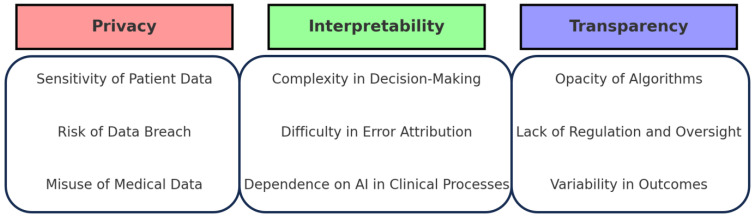
Challenges of Using LLMs in Healthcare.

Large language models like ChatGPT are less accurate in specialized domains like the basics of MR, which is what we studied, and clinical medicine for several reasons, and we need to be aware of this fact. Firstly, these models need a deeper understanding of the meaning and context of the language they generate, as they rely on statistical patterns and word associations [14]. This lack of understanding can lead to the production of factually incorrect statements and the overlooking of crucial medical findings [15]. Additionally, these models may recommend unnecessary investigations and overtreatment, which can be potentially harmful [16]. Furthermore, the responses generated by these models can vary inconsistently with repeat queries, indicating a need for more stability in their performance [17]. Lastly, these models’ fallibility and the difficulty distinguishing their responses from human answers pose potential threats to teaching and learning in healthcare education [18]. Therefore, whereas large language models have potential in healthcare, their limitations and risks must be carefully considered before widespread use in specialized domains like healthcare. Understanding and addressing these challenges is critical for healthcare professionals. The following schematic illustrates the key takeaways (Figure 4).

**Figure 5:**
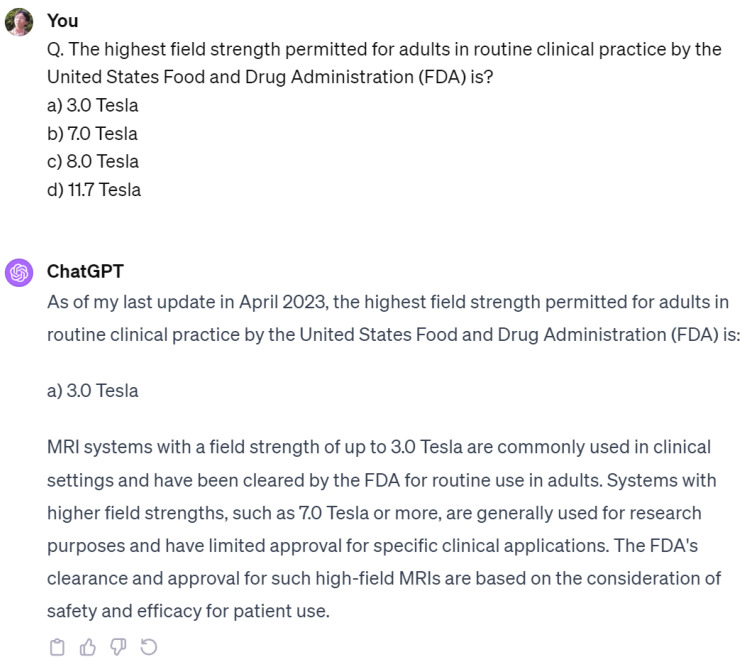
Example of an incorrect answer given by ChatGPT. ChatGPT gave (a) 3.0 Tesla as the correct answer. However, the correct answer is (c) 8.0 Tesla. (The current FDA limit for clinical MR scanners is 8.0 T for adults and children older than 1 month; it is 4.0 T for children younger than 1 month).

In our study, ChatGPT needs to improve in solving multiple-choice questions (MCQs) related to the medical field (Figure 5). MCQs often require a deeper understanding of the context, which is challenging for machine learning algorithms. Additionally, the complex answer options with subtle differences in medical MCQs make it difficult for the algorithm to differentiate accurately. The presence of distracting or irrelevant information in MCQs can lead to interpretation by the algorithm, resulting in accurate answers [19,20,21].

The findings of our study reveal a significant disparity in ChatGPT’s performance between answering straightforward MRI questions and tackling specialized multiple-choice questions. This variance underscores the complexities inherent in applying AI in medical education and clinical decision-making. ChatGPT’s proficiency in handling basic questions suggests it can be a valuable tool for educating medical students and junior radiologists. However, its limitations in solving complex, case-based scenarios indicate a need for cautious integration of AI in clinical practice, especially in diagnostic processes.

AI models, such as ChatGPT, have limitations in context-heavy and specialized tasks like radiology. These limitations are particularly pronounced in fields that require nuanced interpretation and detailed anatomical knowledge. To bridge the gap between general knowledge dissemination and specialized case handling, it is crucial to enhance AI models’ capabilities in context awareness and depth of medical knowledge. This study emphasizes the need for AI models that not only process text but also understand the underlying medical concepts [19,22].

Furthermore, the need for high-quality labeled training data is a significant limitation in using machine learning algorithms in the medical domain. Obtaining labeled data in the medical field is costly, leading to biased, incomplete, or insufficiently diverse datasets for training medical models. This results in suboptimal performance of the models [23]. Additionally, models trained on labeled data may struggle when faced with new data that differ significantly from the training data, known as a domain shift. The medical domain is particularly prone to domain shifts due to the wide variation in patients’ clinical presentations and conditions [24].

ChatGPT has demonstrated high accuracy in generating responses to text input thanks to its ability to recognize and utilize abstract relationships between words within a neural network [25]. It is effective for answering general or straightforward questions and generating coherent and contextually appropriate responses [5]. ChatGPT has a Language Learning Module (LLM) that allows it to continually learn and improve its performance by adapting to new data and contexts [26].

ChatGPT has impressive capabilities but should not be overly relied upon in specialized healthcare, particularly in radiology. It has limitations in terms of needing more specific knowledge and experience in radiology [25]. ChatGPT’s accuracy is dependent on the data it has been trained on, which may need revision for specialized medical applications [27]. Furthermore, ChatGPT cannot understand the complexity of medical images or interpret their significance, which is crucial in radiological interpretation [28]. As a result, ChatGPT’s answers may need to be improved in usefulness and accuracy for radiological diagnosis and treatment planning [29].

To enhance AI performance in complex medical fields, incorporating diverse and extensive datasets, including complex case studies and advanced imaging interpretations, could be beneficial [2]. Integrating AI models with clinical decision support systems may offer a more comprehensive understanding and improved accuracy in complex scenarios [30]. Collaborations between AI developers and medical professionals are essential in creating datasets that reflect the intricacies of real-world medical cases [31].

MRI basics and physics are essential for the practice of radiology and can be used for clinical purposes. Understanding the principles of MRI physics, signal generation, and image contrast mechanisms is crucial for non-radiology clinicians to interpret MR images and facilitate interdisciplinary understanding [32]. Some resources, such as textbooks, provide a conceptual approach to understanding the basics of MRI from both clinical and technological perspectives. However, in anticipation of the many uses of large language models, ChatGPT can be used partly for educational purposes on MRI basics by providing concise and easy-to-understand explanations without requiring extensive technical background. In our study, ChatGPT showed high accuracy on simple, short-answer questions about MRI physics (observer 1, 86% correct; observer 2, 88% correct). However, based on the generally inaccurate results for multiple-choice questions, it may stretch it to use ChatGPT for clinical medicine education, including MRI physics, without validation.

In fact, from its inception, ChatGPT has been used by the public and medical professionals, including professors and residents, for problem-solving and obtaining information. However, its use in medical education and practice should be approached cautiously due to its nature as a language generation model and its accuracy in specialized medical areas. While ChatGPT offers potential applications in medical education, such as generating exercises, quizzes, and scenarios for students to practice and evaluate their understanding of medical concepts, there are challenges and limitations to consider. These include the need to carefully assess the accuracy and reliability of ChatGPT responses, address its limitations in understanding medical terminology and context, and consider ethical concerns regarding patient privacy. Despite the potential benefits, further research is needed to effectively integrate ChatGPT into medical education and explore its impact on learning outcomes, critical thinking skills, and student and faculty satisfaction [33].

Moreover, the integration of AI in healthcare raises ethical and practical considerations. Patient safety and data privacy are paramount, and stringent quality checks and human oversight are necessary to ensure the reliability of AI responses [34]. Continuous updates and learning for AI systems are also crucial due to the evolving nature of medical knowledge and practice [35]. Establishing guidelines and protocols for the safe and effective use of AI in healthcare is essential as it becomes more prevalent [36].

The limitations of our study are below. First, a few researchers conducted the study at only a single institution, so it generated little question input. Second, although we obtained offline multiple-choice questions from the public open website (Courtesy of Allen D. Elster, MRIquestions.com), we could only obtain a few questions due to the specificity and scope of MRI. This problem can be solved in the future if multiple researchers submit many questions for input or if a more extensive set of publicly available questions is obtained.

This study opens several avenues for future research. Exploring the integration of visual data, such as MRI scans, with textual data could enhance AI’s diagnostic capabilities. Longitudinal studies involving a more extensive set of questions and diverse clinical scenarios could provide deeper insights into AI’s applicability in radiology. Additionally, comparative studies involving different AI models could determine the most effective approaches in medical AI.

## 4. Conclusions

We report the accuracy of ChatGPT in the MRI domain and found that, while ChatGPT was generally accurate in answering simple, well-defined questions, it needed improvement in solving off-the-beaten-path multiple-choice questions that required specialized MR knowledge and judgment. The study also highlighted the limitations of ChatGPT in specialized healthcare, especially radiology, due to its need for more specific knowledge and experience in radiology and its inability to understand the complexity of medical images or interpret their significance, which is crucial in radiological interpretation. We emphasized that ChatGPT should be balanced in specialized healthcare and that its use in medical education and practice should be considered cautiously. However, ChatGPT demonstrated significant potential in the healthcare domain as a tool for generating natural language responses to medical questions and assisting healthcare professionals in decision-making, potentially improving patient outcomes and increasing efficiency in healthcare settings. We also discussed the limitations of using machine learning algorithms, including ChatGPT, in the medical domain, such as the need for high-quality labeled training data and the issue of domain shift. Overall, the study underscores the need for continued research and development to improve the accuracy and applicability of language generation models like ChatGPT in specialized healthcare.

## Figures and Tables

**Table 1:** Result of simple questions about magnetic resonance imaging.

(Q = 50)	Observer 1	Observer 2
Correct	43 (86%)	44 (88%)
Semi-correct	5 (10%)	5 (10%)
Incorrect	2 (4%)	1 (2%)
Interobserver agreement	0.87 (near perfect)	

**Table 2:** Result of multiple-choice questions related to MRI.

(Q = 15)	Basic Electromagnetism	MR Magnets	Gradients	RF and Coils	Site Planning
Correct	10 (66.7%)	8 (53.3%)	7 (46.7%)	9 (60%)	6 (40%)
Incorrect	5 (33.3%)	7 (46.7%)	8 (53.3%)	6 (40%)	9 (60%)
Accuracy	66.7%	53.3%	46.7%	60%	40%

## Data Availability

The datasets generated and analyzed during the current study are not publicly available due [Consider that the answer may change depending on the version of ChatGPT and the context of the response] but are available from the corresponding author on reasonable request.

## Supplementary Materials

The following supporting information can be downloaded at: https://www.mdpi.com/article/10.3390/diagnostics14020171/s1, Text S1: Single and multiple-choice questions about MRI fundamentals and physics asked on ChatGPT.

## References

[1] Nagi F., Salih R., Alzubaidi M., Shah H., Alam T., Shah Z., Househ M. (2023). Applications of Artificial Intelligence (AI) in Medical Education: A Scoping Review.

[2] Mehta V. (2023). Artificial Intelligence in Medicine: Revolutionizing Healthcare for Improved Patient Outcomes. J. Med. Res. Innov..

[3] Ghayda R.A., Cannarella R., Calogero A.E., Shah R., Rambhatla A., Zohdy W., Kavoussi P., Avidor-Reiss T., Boitrelle F., Mostafa T. (2024). Artificial Intelligence in Andrology: From Semen Analysis to Image Diagnostics. World J. Men’s Health.

[4] Chakraborty S.M., Chopra H.M., Akash S.M., Chakraborty C., Dhama K.M. (2023). Advances in artificial intelligence (AI)-based diagnosis in clinical practice—Correspondence. Ann. Med. Surg..

[5] Harry A. (2023). The Future of Medicine: Harnessing the Power of AI for Revolutionizing Healthcare. Int. J. Multidiscip. Sci. Arts.

[6] del Rio-Chanona M., Laurentsyeva N., Wachs J. (2023). Are Large Language Models a Threat to Digital Public Goods? Evidence from Activity on Stack Overflow. arXiv.

[7] Mago J., Sharma M. (2023). The Potential Usefulness of ChatGPT in Oral and Maxillofacial Radiology. Cureus.

[8] Srivastav S., Chandrakar R., Gupta S., Babhulkar V., Jaiswal A., Prasad R., Wanjari M.B., Agarwal S. (2023). ChatGPT in Radiology: The Advantages and Limitations of Artificial Intelligence for Medical Imaging Diagnosis. Cureus.

[9] Bhayana R., Krishna S., Bleakney R.R. (2023). Performance of ChatGPT on a Radiology Board-style Examination: Insights into Current Strengths and Limitations. Radiology.

[10] Mohamadi S., Mujtaba G., Le N., Doretto G., Adjeroh D.A. (2023). ChatGPT in the Age of Generative AI and Large Language Models: A Concise Survey. arXiv.

[11] Orrù G., Piarulli A., Conversano C., Gemignani A. (2023). Human-like problem-solving abilities in large language models using ChatGPT. Front. Artif. Intell..

[12] D’antonoli T.A., Stanzione A., Bluethgen C., Vernuccio F., Ugga L., Klontzas M.E., Cuocolo R., Cannella R., Koçak B. (2023). Large language models in radiology: Fundamentals, applications, ethical considerations, risks, and future directions. Diagn. Interv. Radiol..

[13] Lee K.H., Lee R.W., Kwon Y.E. (2023). Validation of a Deep Learning Chest X-ray Interpretation Model: Integrating Large-Scale AI and Large Language Models for Comparative Analysis with ChatGPT. Diagnostics.

[14] Woodland T. (2023). ChatGPT for Improving Medical Education: Proceed with Caution. Mayo Clin. Proc. Digit. Health.

[15] Zhang J., Sun K., Jagadeesh A., Ghahfarokhi M., Gupta D., Gupta A., Gupta V., Guo Y. (2023). The Potential and Pitfalls of using a Large Language Model such as ChatGPT or GPT-4 as a Clinical Assistant. arXiv.

[16] Beaulieu-Jones B.R., Shah S., Berrigan M.T., Marwaha J.S., Lai S.L., Brat G.A. (2023). Evaluating Capabilities of Large Language Models: Performance of GPT4 on American Board of Surgery Qualifying Exam Question Banks. medRxiv.

[17] Davies N.P., Wilson R., Winder M.S., Tunster S.J., McVicar K., Thakrar S.T., Williams J., Reid A. (2023). ChatGPT sits the DFPH exam: Large language model performance and potential to support public health learning. medRxiv.

[18] Meskó B., Topol E.J. (2023). The imperative for regulatory oversight of large language models (or generative AI) in healthcare. npj Digit. Med..

[19] Mishra A., Begley S.L., Chen A., Rob M., Pelcher I., Ward M., Schulder M. (2023). Exploring the Intersection of Artificial Intelligence and Neurosurgery: Let us be Cautious with ChatGPT. Neurosurgery.

[20] Beilby K., Hammarberg K. (2023). O-089 Using ChatGPT to answer patient questions about fertility: The quality of information generated by a deep learning language model. Hum. Reprod..

[21] Ali R., Tang O.Y., Connolly I.D., Fridley J.S., Shin J.H., Sullivan P.L.Z., Cielo D., Oyelese A.A., Doberstein C.E., Telfeian A.E. (2023). Performance of ChatGPT, GPT-4, and Google Bard on a Neurosurgery Oral Boards Preparation Question Bank. Neurosurgery.

[22] Sarbay İ., Berikol G.B., Özturan İ.U. (2023). Performance of emergency triage prediction of an open access natural language processing based chatbot application (ChatGPT): A preliminary, scenario-based cross-sectional study. Turk. J. Emerg. Med..

[23] Huang Z., Jiang R., Aeron S., Hughes M.C. (2023). Accuracy versus time frontiers of semi-supervised and self-supervised learning on medical images. arXiv.

[24] Zhang D., Zhong C., Guo Y., Hong Y., Zhang J. (2023). MetaHead: An Engine to Create Realistic Digital Head. arXiv.

[25] Biswas S., Logan N.S., Davies L.N., Sheppard A.L., Wolffsohn J.S. (2023). Assessing the utility of ChatGPT as an artificial intelligence-based large language model for information to answer questions on myopia. Ophthalmic Physiol. Opt..

[26] Bagno E., Dana-Picard T., Reches S. (2023). ChatGPT may excel in States Medical Licensing Examination but falters in basic Linear Algebra. arXiv.

[27] Meo S.A., Al-Masri A.A., Alotaibi M., Meo M.Z.S., Meo M.O.S. (2023). ChatGPT Knowledge Evaluation in Basic and Clinical Medical Sciences: Multiple Choice Question Examination-Based Performance. Healthcare.

[28] Scanlon M., Breitinger F., Hargreaves C., Hilgert J.N., Sheppard J. (2023). ChatGPT for Digital Forensic Investigation: The Good, the Bad, and the Unknown. Forensic Sci. Int. Digit. Investig..

[29] Abouammoh N., Alhasan K., Raina R., Malki K.A., Aljamaan F., Tamimi I., Muaygil R., Wahabi H., Jamal A., Al-Tawfiq J.A. (2023). Exploring Perceptions and Experiences of ChatGPT in Medical Education: A Qualitative Study Among Medical College Faculty and Students in Saudi Arabia. medRxiv.

[30] Arasteh S.T., Lotfinia M., Nolte T., Saehn M., Isfort P., Kuhl C., Nebelung S., Kaissis G., Truhn D. (2023). Preserving privacy in domain transfer of medical AI models comes at no performance costs: The integral role of differential privacy. arXiv.

[31] Nyberg C.C., Morris E. (2023). Letter to the editor: “Revolutionizing clinical education: Opportunities and challenges of AI integration”. Eur. J. Physiother..

[32] Azhar S., Chong L.R. (2022). Clinician’s guide to the basic principles of MRI. Heart.

[33] Liu J., Liu S. (2023). The application of ChatGPT in medical education. EdArXiv.

[34] Al-Hwsali A., Alsaadi B., Abdi N., Khatab S., Alzubaidi M., Solaiman B., Househ M. (2023). Scoping Review: Legal and Ethical Principles of Artificial Intelligence in Public Health. Stud. Health Technol. Inform..

[35] Amedior N.C. (2023). Ethical Implications of Artificial Intelligence in the Healthcare Sector. Adv. Multidiscip. Sci. Res. J. Publ..

[36] Al Kuwaiti A., Nazer K., Al-Reedy A., Al-Shehri S., Al-Muhanna A., Subbarayalu A.V., Al Muhanna D., Al-Muhanna F.A. (2023). A Review of the Role of Artificial Intelligence in Healthcare. J. Pers. Med..

