# Supplementary material for: ChatGPT’s Accuracy on Magnetic Resonance Imaging Basics: Characteristics and Limitations Depending on the Question Type
Source: Diagnostics (Basel). 2024 Jan 12;14(2):171. doi: 10.3390/diagnostics14020171 (PMC10814518; doi:10.3390/diagnostics14020171)
Supplement: Supplementary file 1 [file diagnostics-14-00171-s001.zip › diagnostics-2782381-supplementary.pdf]

Single Choice questions

- **Courtesy of Ro Woon Lee, Kyu Hong Lee**

1. What is the first company to produce a clinical whole-body MRI scanner for commercial use?
2. Describe the function of the main magnet in an MRI machine.
3. Explain the role of gradient coils in MRI.
4. What are RF coils, and why are they important in MRI?
5. Define T1 relaxation and its significance in MRI.
6. Define T2 relaxation and how it differs from T1 relaxation.
7. Explain the concept of proton density in MRI.
8. Describe the phenomenon of magnetic resonance.
9. What is the Larmor equation, and why is it important in MRI?
10. Explain the process of signal acquisition in MRI.
11. What is a Fourier Transform, and how is it used in MRI?
12. Describe the concept of k-space in MRI imaging.
13. What is meant by MRI contrast, and how is it achieved?
14. Explain the difference between T1-weighted and T2-weighted images.
15. What are the safety concerns associated with MRI?
16. How does field strength affect MRI image quality?
17. What is a spin-echo sequence, and how does it work?
18. Define the term "echo time" (TE) in MRI.
19. What is the significance of repetition time (TR) in MRI?
20. Explain the concept of inversion recovery in MRI.
21. How do magnetic field inhomogeneities affect MRI images?
22. Describe the phenomenon of chemical shift in MRI.
23. What is fat suppression in MRI, and how is it achieved?
24. Explain the principles of diffusion-weighted imaging (DWI).
25. What is functional MRI (fMRI), and how does it work?
26. Describe the concept of magnetic susceptibility in MRI.
27. How does gadolinium contrast enhance MRI images?
28. What is the principle behind MR angiography?
29. Explain the differences between 1.5T and 3T MRI scanners.
30. How does echo-planar imaging (EPI) work in MRI?
31. Describe the safety considerations for MRI-compatible devices.
32. What is the role of SAR (Specific Absorption Rate) in MRI safety?
33. How does temperature affect MRI imaging and safety?
34. Explain the importance of coil sensitivity in MRI.
35. What is parallel imaging, and how does it improve MRI?
36. Describe the role of phase encoding in MRI.

37. How is spatial resolution determined in MRI?
38. What are the causes and effects of artifacts in MRI images?
39. Explain the concept of time of flight (TOF) in MR angiography.
40. How do metal implants affect MRI imaging?
41. What is the significance of the Nyquist theorem in MRI?
42. Describe the principle of phase contrast MR angiography.
43. How do pre-pulses enhance MRI imaging?
44. What is the impact of relaxation times on image contrast in MRI?
45. Explain the process of slice selection in MRI.
46. How does motion affect MRI image quality, and how is it managed?
47. What is the principle of cardiac gating in MRI?
48. Describe the application of MRI in neuroimaging.
49. How is MRI used in musculoskeletal imaging?
50. What is the highest magnetic field strength that the U.S. Food and Drug Administration (FDA) allows adults in routine clinical practice?

# Multi-choice questions

– Courtesy of Allen D. Elster, MRIquestions.com

Basic Electromagnetism

1. When the current flowing through a wire reverses direction, the magnetic field around the wire
    a. Does not change
    b. Increases
    c. Disappears
    d. Reverses direction
2. The bulk magnetic properties of matter derive primarily from
    a. Protons
    b. Neutrons
    c. Electrons
    d. Whole nuclei
3. If the current in a wire doubles, the induced magnetic field
    a. Doubles
    b. Quadruples
    c. Remains the same
    d. Is reduced by half
4. The direction of magnetic field lines surrounding a wire can be determined using
    a. The right-hand rule
    b. The left-hand rule
    c. Faraday's Law
    d. Lenz' Law

5. The voltage induced across a stationary conductor in an external static magnetic field
    a. Depends on the angle of the conductor with the magnetic field
    b. Increases with time
    c. Is zero
    d. Depends on the strength of the magnetic field

6. Concerning the relationship between electricity and magnetism, which of the following statements is false?
    a. A constant current in a wire induces a constant magnetic field around the wire.
    b. A changing current in a wire induces a changing magnetic field around the wire.
    c. A constant magnetic field induces voltage in a nearby stationary wire.
    d. A changing magnetic field induces voltage in a nearby wire.

7. Which question about the Tesla (T) is correct?
    a. It is the official unit for magnetic induction field strength in the cgs system.
    b. 1 Tesla = 1,000 Gauss (G)
    c. 1 G = 1 mT
    d. It is one of the coolest cars on the road

8. Concerning magnetic field strengths, which statement is true?
    a. The earth's magnetic field is about 0.5 G.
    b. A junkyard electromagnet that picks up cars is much stronger than the main field of most MR scanners.
    c. Research MR scanners for humans now exist with field strengths exceeding 20 T.
    d. Higher field strength scanners have wider bores than lower field strength scanners to accommodate the extra flux lines

9. Which of the following materials is paramagnetic?
    a. Water
    b. Fat
    c. Bone
    d. Air

10. A material that is weakly repulsed by a magnetic field is known as
    a. Paramagnetic
    b. Diamagnetic
    c. Superparamagnetic
    d. Ferromagnetic

11. Susceptibility (χ) is negative for materials that are
    a. Paramagnetic
    b. Superparamagnetic
    c. Diamagnetic
    d. Ferromagnetic

12. Ferromagnetic materials form magnetic ________ when arrays of electron spins become linked via quantum exchange interaction.
    a. Flux lines
    b. Poles
    c. Vectors
    d. Domains

13. Comparing superparamagnetic and ferromagnetic materials, which statement is false?
    a. Ferromagnetism is usually more powerful than superparamagnetism.
    b. Ferromagnetism persists when the magnetizing field is removed.
    c. Superparamagnetism persists once the external field is removed.
    d. Superparamagnetism can be thought of as a single-domain particle.

14. During a magnetic quench, why should patients and employees be evacuated from the scan room?
    a. Even in small quantities gaseous helium causes burning and irritation to the eyes.
    b. Asphyxiation may occur.
    c. Severe frostbite would be likely.

   d. The released helium may catch fire or explode.

15. How many sets of paired physical gradients are present in an MR scanner?
    a. 1
    b. 2
    c. 3
    d. 6

MR Magnets

1. The most common design configuration for clinical MR scanners is
    a. Open bore superconducting
    b. Closed bore superconducting
    c. Open bore permanent
    d. Dipolar electromagnet
2. The highest field strength permitted for adults in routine clinical practice by the United States Food and Drug Administration (FDA) is
    a. 3.0 Tesla
    b. 7.0 Tesla
    c. 8.0 Tesla
    d. 11.7 Tesla
3. The first company to produce a clinical whole-body MRI scanner for commercial use was
    a. GE
    b. Fonar
    c. Siemens
    d. Technicare
4. The direction of the main magnetic field (B_o) in a cylindrical closed bore scanner is
    a. Longitudinal (along the main axis) of the cylinder
    b. Horizontal (cross-wise to the cylinder and parallel to the floor)
    c. Vertical (cross-wise to the cylinder and perpendicular to the floor)
    d. Can be at any angle depending on which gradients are turned on
5. Which of the following is not an advantage of low- and intermediate-field (< 1.0 T) MR scanners?
    a. Lower price
    b. Lower fringe field
    c. Improved detection of gadolinium enhancement
    d. Lower energy deposition in tissues
6. Which of the following is not an advantage of high-field (≥ 1.0 T) MR scanners?
    a. Higher signal-to-noise
    b. Better detection of calcifications and hemorrhage
    c. Smaller artifacts around metallic implants
    d. Better magnetic field homogeneity
7. Quoted specifications for four different magnets are given below. Which one has the best homogeneity?
    a. <1 ppm over a 40 cm DSV
    b. <1 ppm over a 20 cm DSV
    c. >1 ppm over a 40 cm DSV
    d. >1 ppm over a 20 cm DSV
8. Poor magnetic field homogeneity may affect image quality in the following ways
    a. Shading artifacts
    b. Spatial distortion
    c. Poor fat suppression
    d. Only a) and b)

    e. All of the above

9. Which of the following statements about passive shimming is true?
    a. Its primary purpose is to correct for field distortions produced by a patient's body.
    b. Ferromagnetic materials cannot be used for passive shimming.
    c. Passive shimming is affected by room temperature.
    d. Once the field is calibrated and magnetic homogeneity achieved, the passive shim materials can be removed.

10. Which of the following statements about superconductivity is correct?
    a. All elements can become superconducting if the temperature is low enough.
    b. Only metals can become superconductors.
    c. The magnetic field is zero inside the center of a superconducting wire.
    d. The resistance of a wire linearly decreases toward zero as the temperature falls below the transition temperature (T_C).

11. The superconducting component in the main windings of nearly all clinical MR scanners is an alloy of
    a. Niobium (Nb) and Titanium (Ti)
    b. Niobium (Nb) and Copper (Cu)
    c. Nickel (Ni) and Titanium (Ti)
    d. Nickel (Ni) and Copper (Cu)

12. The cryostat of a typical superconducting MR scanner contains all of the following except
    a. Liquid helium
    b. Liquid nitrogen
    c. Main magnet windings
    d. superconducting shim coils

13. The temperature of liquid helium is approximately
    a. 4 °K
    b. 0 °K
    c. −4 °K
    d. −400 °C

14. MRI facilities often display a sign on the door that says: "Warning! The magnet is always on." This sign would not strictly apply to a
    a. Resistive magnet scanner
    b. Permanent magnet scanner
    c. Superconducting magnet scanner
    d. The sign is applicable to all types of scanners, always.

15. Pushing the "big red button" near the door to a room housing an MRI scanner
    a. Immediately opens the door even if scanning is in progress
    b. Initiates a controlled quench of the magnetic field
    c. Turns off all electric power to the scanner and room
    d. Calls emergency providers (911) and sounds an alarm

Gradients

1. Magnetic field gradients for imaging are typically measured in units of
    a. Millitesla per meter (mT/m)
    b. Gauss per second (G/s)
    c. Tesla (T)
    d. Tesla per meter per second (T/m-s)

2. For a supine patient, enabling the *z*-gradient alone to alter the magnetic field within a patient during slice selection would create a(n)
    a. Axial slice
    b. Coronal slice

c. Sagittal slice
d. Oblique slice

3. What is the effect of applying the *x*- and *z*-gradients simultaneously during slice selection?
    a. The image will be distorted.
    b. Significant interslice cross-talk will occur.
    c. An oblique slice will be created.
    d. The scanner will display a warning that such a combination is not allowed.
4. When the *y*-gradient is turned on, what happens to the direction of the main ($\mathbf{B_o}$) field?
    a. The $\mathbf{B_o}$ field now points slightly to the right.
    b. The $\mathbf{B_o}$ field now points slightly toward the ceiling.
    c. The $\mathbf{B_o}$ field is reversed.
    d. The $\mathbf{B_o}$ field remains pointing in its original (*z*)-direction.
5. The basic coil configuration used to generate the *z*-gradient in a cylindrical MR scanner is known as
    a. Maxwell pair.
    b. Double saddle.
    c. Golay.
    d. Fingerprint.
6. Which of the following statements about eddy currents is false?
    a. They create a wide range of image artifacts, including ghosts and blurring.
    b. They are a manifestation of Faraday's Law of induction.
    c. They especially affect traditional spin-echo sequences with long *TE's*.
    d. They create tissue heating.
7. Concerning actively shielded gradients, which statement is true?
    a. The are the most effective way to reduce eddy currents in superconducting systems.
    b. The are the most effective way to reduce eddy currents in superconducting systems.
    c. Current in these coils runs in a direction opposite to their associated imaging gradients.
    d. All of the above are true.
8. Which of the following methods is not used to reduce eddy currents:
    a. Actively shielded gradients.
    b. Self-shielded gradients.
    c. Active shimming coils.
    d. Pre-compensation.
9. Typical values for peak gradient strength in a modern 1.5 T scanner are in the range of
    a. 1-10 mT/m.
    b. 20-50 mT/m.
    c. 200-400 mT/m.
    d. 500-1000 mT/m.
10. The time for a gradient to ramp from zero to its maximum value is known as its
    a. Rise time.
    b. Gradient time.
    c. Slew rate.
    d. Duty cycle.
11. The definition of gradient slew rate is
    a. Peak gradient strength ÷ main field strength (*Bo*).
    b. Peak gradient strength ÷ total time the gradient is on.
    c. Peak gradient strength ÷ Rise time.
    d. The number of times a gradient is turned on and off per second.
12. The units for slew rate are given in
    a. Millitesla per meter (mT/m).
    b. Tesla per meter per second (T/m/s).
    c. Tesla per second (T/s).
    d. Milliseconds (ms).

13. Typical maximum slew rate values quoted for modern 1.5 T scanners are in the range of
    a. 1−2 T/m/s.
    b. 10−20 T/m/s.
    c. 100−200 T/m/s.
    d. 1000−2000 T/m/s.
14. A gradient that ramps from 0 to a peak amplitude of 30 mT/m in 0.25 ms has a slew rate of
    a. 30 T/m/s.
    b. 60 T/m/s.
    c. 90 T/m/s.
    d. 120 T/m/s.
15. Which gradient specification is generally the most important when assessing how well an MR system is capable of performing rapid, high-resolution imaging?
    a. Peak gradient strength.
    b. Slew rate.
    c. Rise time.
    d. Magnetic field strength.

RF and Coils

1. Which coils are located closest to the patient in an MR scanner?
    a. Gradient coils.
    b. RF-receiver coils.
    c. Shim coils.
    d. Body RF-transmit coils.
2. In the construction of a superconducting MR magnet, which is the correct order of coils from outermost to innermost?
    a. Main magnet windings, gradient coils, RF coils, shielding coils.
    b. Gradient coils, shield coils, main magnet windings, RF coils.
    c. Shielding coils, main magnet windings, gradient coils, RF coils.
    d. RF coils, shield coils, main magnet windings, gradient coils.
3. Although most local RF coils are "receive only", some specially designed to operate in "transmit-receive (T/R)" mode. T/R coils commonly offered by MR vendors include all of the following except
    a. Head coils.
    b. Knee coils.
    c. Spectroscopy coils.
    d. Spine coils.
4. A 1.5 T MR scanner has a base operating frequency of approximately 64 MHz. In the electromagnetic spectrum, this is considered to be in the range of
    a. Infrared frequencies.
    b. Radio frequencies.
    c. X-ray frequencies.
    d. Microwave frequencies.
5. Use of a single element surface coil placed directly on the patient offers which advantages?
    a. High signal-to-noise.
    b. Increased depth of penetration.
    c. Capability for larger fields-of-view.
    d. All of the above.
6. Comparing 10 cm and 20 cm diameter surface coils, which of the following is false?
    a. The sensitive volume of the 20 cm coil is larger.
    b. The penetration depth of the 20 cm coil is greater.
    c. The 20 cm coil has higher signal-to-noise for voxels immediately under the coil.

d. The 20 cm coil offers a larger field of view.

7. Comparing linear and quadrature coils
   a. Quadrature coils offer twice the signal-to-noise.
   b. Quadrature coils offer four times the signal-to-noise.
   c. Quadrature coils offer about 40% greater signal-to-noise.
   d. Quadrature coils are about 40% larger.

8. A sinusoidal wave can be described by the equation $S(t) = A \sin(\omega t - \varphi)$. The constant A represents
   a. Angular frequency.
   b. Difference in height between positive and negative peaks.
   c. Half the difference in height between positive and negative peaks.
   d. Phase shift.

9. An MR scanner employs three different magnetic fields— the main field ($\mathbf{B_0}$), gradient fields ($\mathbf{G}$), and radiofrequency field ($\mathbf{B_1}$). In terms of relative strength from weakest to strongest, the proper ranking is
   a. $\mathbf{B_1} < \mathbf{G} < \mathbf{B_0}$
   b. $\mathbf{G} < \mathbf{B_0} < \mathbf{B_1}$
   c. $\mathbf{G} < \mathbf{B_1} < \mathbf{B_0}$
   d. $\mathbf{B_1} < \mathbf{B_0} < \mathbf{G}$

10. Which of the following is not an advantage of parallel (multi-)transmit RF?
   a. Decreased RF-energy deposition in tissues.
   b. Reduced shading artifacts.
   c. Increased standing waves due to dielectric effect.
   d. More uniform excitation.

11. Comparing phased array and parallel array coils, which of the following is true?
   a. Both types of coils offer improved signal-to-noise and increased field-of-view.
   b. Overlap of coil elements is avoided in both types.
   c. Phased array coils are also known as switchable arrays.
   d. Both can be used equally well with parallel imaging acquisition methods.

12. Parallel imaging systems are composed of coil elements, segments, and channels. The proper transmission hierarchy beginning with the patient and proceeding upward through the processing chain is
   a. Elements → channels → segments
   b. Elements → segments → channels
   c. Elements → channels → segments
   d. Channels → elements → channels

13. Advantages of parallel receiver coil arrays include all of the following except
   a. Increased signal-to-noise.
   b. Increased field-of-view.
   c. Ease of design.
   d. Reduced imaging time.

14. The effective depth of penetration for signal reception from a 20 cm diameter single loop surface coil is approximately
   a. 10−15 cm
   b. 20−25 cm
   c. 30−40 cm
   d. 40−50 cm

15. Concerning the main transmit RF-body coil, which statement is false?
   a. It is commonly used to receive the MR signal
   b. It is built into the scanner gantry housing and cannot be seen by the patient
   c. It is considered a transceiver coil, capable of both RF transmission and reception.
   d. Its transmission field ($\mathbf{B_1}$) is perpendicular to the main magnetic field ($\mathbf{B_0}$).

SITE PLANNING

1. Which of the following components of an MR system is typically not located in an adjoining equipment room?
    a. RF-power amplifiers
    b. Gradient amplifiers
    c. Helium pump
    d. Gradient coils
2. Where is the master computer located that controls the MR scanner and processes data into images?
    a. In the MR scanner room
    b. In the MR scanner control room
    c. In the nearby MR equipment room
    d. At least 25 meters distant from the main scanner to avoid interference
3. The function of the array processor is to
    a. Generate triggers for the array of RF-pulses and gradient waveforms used for imaging
    b. Reconstruct the raw NMR data into images
    c. Calculate RF frequency offsets and gradient strengths for desired slice selection and field-of-view
    d. Activate and/or disable various coil elements in an array
4. Which scanner is the heaviest (and would thus require the most floor support)?
    a. 0.35 T Permanent magnet system
    b. 0.6 T Resistive magnet system
    c. 1.5 T Superconductive system
    d. 3.0 T Superconductive system
5. Which scanner is would have the lowest overall siting and operational costs?
    a. 0.35 T Permanent magnet system
    b. 0.6 T Resistive magnet system
    c. 1.5 T Superconductive system
    d. 3.0 T Superconductive system
6. Which component of a superconducting MR scanner does not require specialized cooling to maintain function?
    a. Main coil windings
    b. Gradient coils
    c. Gradient amplifiers
    d. Radiofrequency coils
    e. Radiofrequency amplifiers
7. The $\mathbf{B_0}$ field of an MR scanner is most homogeneous at
    a. At the opening (gantry) of the magnet
    b. At bore level about 1 meter directly in front of the magnet
    c. In the middle of the bore at isocenter
    d. On the outside of the magnet immediately against its wall
8. Which scanner would have the largest fringe field?
    a. 0.35 T Permanent magnet system
    b. 0.6 T Resistive magnet system
    c. 1.5 T Superconductive system
    d. 3.0 T Superconductive system
9. If one moves from 1 meter to 2 meters away from a magnet, the fringe field will be reduced by a factor of approximately
    a. $\sqrt{2}$
    b. 2
    c. 4
    d. 8

10. The fringe fields of cylindrical superconducting magnet are highest
    a. In the *x*-direction (transverse and horizontal to the axis bore)
    b. In the *y*-direction (transverse and vertical to the axis bore)
    c. In the *z*-direction (along the axis bore)
    d. They are equal in all directions

11. The primary purpose for passive magnetic shielding is
    a. To reduce fringe magnetic fields outside the scanner room.
    b. To keep extraneous radiofrequency noise from entering the scanner room.
    c. To constrain the NMR signal to remain within the bore of the magnet for better reception.
    d. To reduce the effects of moving equipment (such as cars and elevators) from distorting the magnetic field.

12. Concerning passive shielding, which statement is true?
    a. It is performed by placing heavy copper plates along the walls of the scanner room.
    b. It is a method to reduce extraneous radiofrequency interference with the MR signal.
    c. It is more commonly required for 7.0T than for 1.5 T installations.
    d. Active shielding technology found in modern scanner design has not changed the need for it.

13. Passive magnetic shielding of the scanner room is typically achieved using sheets or rods made of
    a. Copper
    b. Iron
    c. Aluminum
    d. Lead

14. The fringe magnetic field arising from an MR scanner
    a. Can be eliminated by active shielding.
    b. Can be eliminated by passive shielding.
    c. Can be reduced by radiofrequency shielding.
    d. None of the above.

15. What is the “5 Gauss Line”?
    a. A place inside the scanner where *x*- and *y*-gradients differ in strength by less than 5 Gauss (5 mT).
    b. The boundary in an MRI center inside of which one’s credit cards will be erased.
    c. A fringe field line that may pose danger to patients with certain pacemakers
    d. A fringe field line in the scanner room safe for patients but which MR technologists should avoid crossing.
